# Supplementary material for: Study on the impact of Kinesiophobia after Total Knee Arthroplasty on the rehabilitation of patients during hospitalization: A pilot study
Source: PLoS One. 2025 Jan 29;20(1):e0317774. doi: 10.1371/journal.pone.0317774 (PMC11778713; doi:10.1371/journal.pone.0317774)
Supplement: Attachment — Submitted filename: Response to reviewers.docx [file pone.0317774.s002.docx]

Dear Editors and reviewers:

Thank you for your letter and for the comments concerning our manuscript entitled”Study on the impact of Kinesiophobia after Total Knee Arthroplasty on the rehabilitation of patients during hospitalization”(PONE-D-24-30449).we have studied comments carefully and have made correction which hope meet with approval.The main corrections in the paper and the responds to the editor and reviewer’s comments are as following:

1.The study has interesting analyses and I will invite independent reviewers to review it, but first please provide the database with the data in unanalyzed values (i.e. absolute values) as a supplementary file (Excel and English).

**Response:**Thank you for your affirmation.We upload our dataset as a supplementary file. We hope that the review experts will point out our statistical deficiencies and give us valuable suggestions.

2.When submitting your revision, we need you to address these additional requirements.Please ensure that your manuscript meets PLOS ONE's style requirements, including those for file naming.

**Response:**Thank you for your kind reminder.We carefully read the format of the magazine and tried our best to make modifications in terms of font size, layout, chart format, reference format, etc. in accordance with the requirements of the magazine. Details can be found in our manuscript. It is inevitable that there are still imperfections, please point them out in time.

3.While revising your submission, please upload your figure files to the Preflight Analysis and Conversion Engine (PACE) digital diagnostic tool, [https://pacev2.apexcovantage.com/](https://c05y1x9s.r.us-east-2.awstrack.me/L0/https://pacev2.apexcovantage.com//1/010f01914bff6f49-9119e034-2ec7-4179-b9e7-25a6012f3803-000000/HVejescMP-IrsZa8oFfUSbxUFf8=172). PACE helps ensure that figures meet PLOS requirements.

**Response:**Before resubmitting our figures,we have uploaded our figure files to the Preflight Analysis and Conversion Engine (PACE) digital diagnostic tool, ensure that figures meet PLOS requirements.

Thanks again to all editors and reviewers for their comments on revisions. We look forward to hearing from you.

Best wishes

Yichao Yao 19 August 2024
