# Supplementary material for: Study on the impact of Kinesiophobia after Total Knee Arthroplasty on the rehabilitation of patients during hospitalization: A pilot study
Source: PLoS One. 2025 Jan 29;20(1):e0317774. doi: 10.1371/journal.pone.0317774 (PMC11778713; doi:10.1371/journal.pone.0317774)
Supplement: Attachment — Submitted filename: Response to Reviewers.docx [file pone.0317774.s004.docx]

**Dear Editors and Reviewers:**

Thank you for your letter and for the reviewers’ comments concerning our manuscript entitled “Study;the impact of Kinesiophobia after Total Knee Arthroplasty on the rehabilitation of patients during hospitalization” (ID:PONE-D-24-30449R1). Those comments are all valuable and very helpful for revising and improving our paper, as well as the important guiding significance to our researches. We have studied comments carefully and have made correction which we hope meet with approval. Revised portion are marked highlight in the paper. The main corrections in the manuscript and the responds to the reviewer’s comments are as flowing: Responds to the reviewer’s comments:

1. **comment：**Include the study design in the title;

**Response:**Thank you very much for your valuable comment.Given our sample size and method of subject selection, we classify our study as “A Pilot Study”.This is our revised title.”Study on the impact of Kinesiophobia after Total Knee Arthroplasty on the rehabilitation of patients during hospitalization：A Pilot Study

”

**2.comment:** Include keywords according to MeSH terms

**Response:**Thank you very much for your valuable comment.We have modified our keywords in accordance with the requirements of meSH terms.This is our modified keyword.”Kinesiophobia Total Knee Arthroplasty Rehabilitation

“

**3.comment:** The objective stated in your abstract is different from the objective stated in your introduction. Please correct this. The sentence describing the objective must be exactly the same in both;

**Response:**Thank you very much for your valuable comment.We have revised the research objectives in the abstract and introduction to ensure they are fully consistent.

**4.comment:**In the last paragraph of the introduction, state a hypothesis;

**Response:**Thank you very much for your valuable comment.As you requested, we state our hypotheses in the last paragraph of the introduction.The following is the hypothesis section we added：

“Based on the preceding discussion and existing research, we propose the following hypotheses. Hypothesis 1: At various measurement time points post-surgery, patients with kinesiophobia will exhibit higher resting pain scores than those without kinesiophobia. Hypothesis 2: At various measurement time points post-surgery, patients with kinesiophobia will demonstrate poorer knee joint flexion compared to those without kinesiophobia. Hypothesis 3: Compared to patients without kinesiophobia, those with kinesiophobia will experience a delayed time to ambulate for the first time after surgery, engage in shorter durations and distances of activity, and report higher pain scores during their initial mobilization. Furthermore, patients with kinesiophobia are expected to have longer postoperative hospitalization durations and perform worse on the two-minute walk test on the day of discharge.

”

**5.comment:**Adjust the methods into topics according to the study design ([https://www.equator-network.org/](https://www.equator-network.org/)). In addition, in the methods section, include a topic called "Sample Size" and explain in detail the sampling strategies used in this study. The statistical analysis part should not include sampling;

**Response:**Thank you very much for your valuable comment.At your request, we have added a topic “Sample Size” to the Methods section and, in the first paragraph of the research process, elaborated on how we sampled our study subjects.The following is the part we added:

**“Sample size**

Sample size calculation was performed using power analysis and sample size (PASS) software (version 2021).The pain scores of the research object were used as the observed outcome indicators. The value of alpha was set as 0.05, power was set as 0.9, number of repeated measurements set as 6, and according to previous literature[9], means was set as 3, standard deviation was set as 3, conditional correlation coefficient set as 0.5.The result showed that one group was expected to recruit 31 patients.”

**“Research process**

This study has been approved by the Ethics Committee of Baoding No.1 Central Hospital. Informed consent forms were signed by all participating patients. In the sampling process of this study, given the constraints of limited research resources and time, we employed a convenience sampling strategy. Specifically, during the study period, investigators assessed whether patients admitted to the hospital met the inclusion criteria by reviewing the medical record system. If a patient satisfied the inclusion criteria, the investigator would bring the informed consent form to the patient’s bedside, provide a brief summary of the study's objectives, and explain the detailed research process. The patient would also be informed of their right to withdraw from the study at any time. Should the patient agree to participate, they would be required to sign the informed consent form. Conversely, if a patient declined to participate, they were assured that this decision would not negatively affect their treatment during hospitalization.

”

**6.comments:**Include the conclusion as a short-topic (describe it in 1 paragraph). The conclusion described in the abstract and the conclusion described after the discussion must be exactly the same.

**Response:**Thank you very much for your valuable comment.We have streamlined our conclusion sections and made sure they are consistent across the conclusion and abstract sections.The following is our revised conclusion section:

“**Conclusion**

In short,compared to patients without kinesiophobia, the presence of kinesiophobia after TKA surgery significantly impacts the efficacy of early rehabilitation exercises during hospitalization. This impact is observed in pain perception, knee joint mobility, the 2-minute walk test ,etc. Early identification of patients with kinesiophobia after TKA and timely intervention are necessary and beneficial.”

We tried our best to improve the manuscript and made some changes in the manuscript. These changes will not influence the content and framework of the paper. We appreciate for Editors/Reviewers’ warm work earnestly, and hope that the correction will meet with approval.

Once again, thank you very much for your comments and suggestions.We look forward to hearing from you as soon as possible.

Kind regards

Yichao Yao

24/12/2024
